# Crystal structure of Sr_5_Te_4_O_12_(OH)_2_, the first basic strontium oxotellurate(IV)

**DOI:** 10.1107/S2056989016015577

**Published:** 2016-10-07

**Authors:** Matthias Weil, Mahdi Shirkhanlou

**Affiliations:** a Institute for Chemical Technologies and Analytics, Division of Structural Chemistry, Vienna University of Technology, Getreidemarkt 9/164-SC, A-1060 Vienna, Austria

**Keywords:** crystal structure, strontium tellurite, channel structure, stereoactive electron lone pair

## Abstract

The asymmetric unit of the title basic strontium oxotellurate(IV), Sr_5_Te_4_O_12_(OH)_2_ {systematic name penta­strontium tetra­kis­[oxotellurate(IV)] di­hydroxide}, comprises three Sr^II^ cations (one with site symmetry 2) and two Te^IV^ atoms, as well as seven O atoms. The coordination numbers of the alkaline earth cations to nearby O atoms range from seven (2 ×) to eight, and the Te^IV^ atoms are surrounded by three oxygen partners in the form of trigonal pyramids. The SrO_*x*_ polyhedra share corners and edges to build up a three-dimensional framework structure encapsulating channels propagating along [010]. The Te^IV^ atoms flank the framework O atoms and are situated at the outer array of the channels with the 5*s* ^2^ lone electron pairs protruding into the empty space of the channels (diameter ≃ 4 Å). Although the H atom of the OH group could not be located, bond-valence-sum calculations and typical O⋯O distances (range 2.81–3.06 Å) clearly indicate hydrogen bonding of medium to weak strengths.

## Abstract 2

In the crystal structure of the basic strontium oxotellurate(IV), Sr_5_Te_4_O_12_(OH)_2_, the principal building blocks, namely SrO_*x*_ polyhedra (*x* = 7 or 8) and trigonal–pyramidal TeO_3_ units are linked into a framework structure delimiting channels parallel to [010].

## Chemical context   

The peculiar feature of the crystal chemistry of oxotellurates(IV) (Christy *et al.*, 2016 ▸) is the presence of the 5*s* ^2^ lone electron pair, denoted *E.* In the majority of cases, the lone electron pair *E* is stereochemically active, making oxotellurates(IV) inter­esting for crystal engineering, *e.g*. in terms of the synthesis of compounds with non-centrosymmetric structures or structures with polar directions. Next to the influence of the (metal) cation on the physico-chemical characteristics of oxotellurates(IV), physical and underlying structural properties of such compounds can also be varied by incorporation of other oxoanions into the oxotellurate(IV) framework, *e.g*. by *p*-block oxoanions such as nitrate (Stöger & Weil, 2013 ▸) or selen­ate (Weil & Shirkanlou, 2015 ▸), or by *d*-block oxoanions such as vanadate (Weil, 2015 ▸).

In this context we attempted the hydro­thermal synthesis of new oxotellurate phases in the system Sr–Te–Se–O–(H). In comparison with typical solid-state reactions using open crucibles under atmospheric conditions, this method is more feasible because Te^IV^ then tends not to be oxidized or to be evaporated during the reaction process. However, a clear disadvantage of the hydro­thermal method is the high(er) number of adjustable parameters (pressure, concentration, temperature, time, filling degree, solvent *etc*), which often makes the products of these experiments difficult to predict or even to reproduce, accompanied by formation of several solid phases in one batch. This was also the case for the present study. Instead of a strontium oxoselenatotellurate, several oxotellurate phases were obtained without incorporation of selenium. Amongst these phases, the title compound, Sr_5_Te_4_O_12_(OH)_2_, a hitherto unknown strontium oxotellurate, was isolated and structurally determined by single crystal X-ray diffraction.

## Structural commentary   

The asymmetric unit of Sr_5_Te_4_O_12_(OH)_2_ comprises three Sr, two Te and seven O atoms (H atoms were not included in the final model, see Section 5 and discussion below). Except one Sr atom (Sr2) that is located on a twofold rotation axis, all atoms are in general positions.

**Figure 1:**
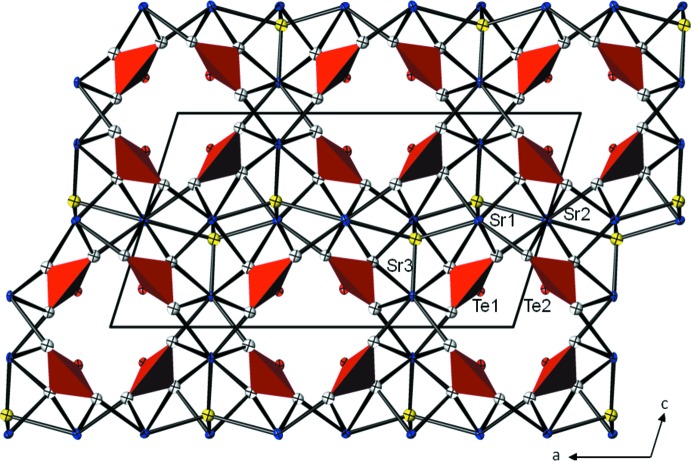
Projection of the crystal structure of Sr_5_Te_4_O_12_(OH)_2_ along [010], with displacement ellipsoids drawn at the 74% probability level. The trigonal–pyramidal TeO_3_ groups are given in red; the O atom representing the OH group is given in yellow, all other O atoms are colourless.

The coordination numbers of the Sr atoms are 7 (for Sr1 and Sr3) and 8 (for Sr2) if Sr—O distances < 3.0 Å are considered as relevant for the first coordination sphere. The corresponding polyhedra are considerably distorted, with Sr—O bond lengths ranging from 2.393 (11) to 2.960 (11) Å (Table 1 ▸) and might be described as monocapped octa­hedra for Sr1 and Sr3, and as a bicapped trigonal prism for Sr2. The SrO_8_ and the two SrO_7_ polyhedra share corners and edges, thereby constructing a three-dimensional framework structure encapsulating channels that propagate along [010]. Each of the two Te atoms connect to the outer oxygen atoms of the framework in a very similar trigonal-prismatic configuration (Table 1 ▸), with the 5*s* ^2^ lone electron pair *E* being stereochemically active, *i.e*. pointing towards the empty space of the channels (Fig. 1 ▸). The channel diameter (without contribution of the lone pairs) is ≃ 4 Å. Te—O bond lengths [1.865 (11)–1.890 (12) Å for Te1 and 1.858 (11)–1.886 (11) Å for Te2] and O—Te—O angles [98.0 (5)–100.3 (5)° for Te1 and 98.8 (5)—101.1 (5)° for Te2] are typical for oxotellurate(IV) anions with three oxygen partners (Christy *et al.*, 2016 ▸).

**Figure 2:**
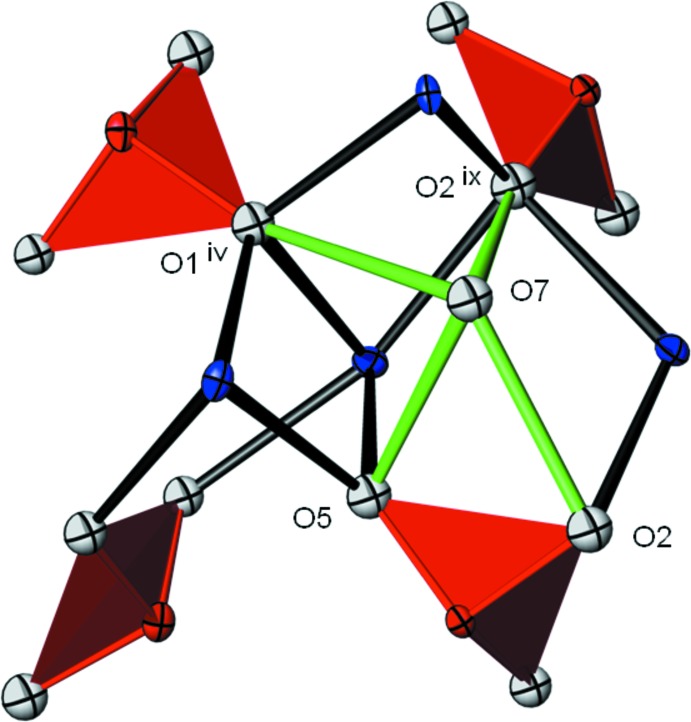
The vicinity of the OH group emphasizing the different possibilities for O⋯O hydrogen bonding (green lines). Sr—(OH) bonds have been omitted for clarity. Symmetry operators refer to those of Table 3 ▸; displacement ellipsoids are the same as in Fig. 1 ▸.

Bond-valence calculations (Brown, 2002 ▸) clearly reveal the presence of an OH group for atom O7 (Table 2 ▸), also required by charge neutrality. Atom O7 is bonded to four Sr atoms (Table 1 ▸, Fig. 1 ▸) and has also four possible oxygen acceptor atoms for hydrogen bonding of medium to weak strength (Table 3 ▸). The situation of four possible acceptor atoms is displayed in Fig. 2 ▸ and makes it appear likely that the corres­ponding H atom of the OH group is positionally disordered and thus could not be located during the present study.

In the sense of a crystal-chemically more detailed formula, the title compound may alternatively be formulated as 4SrTeO_3_·Sr(OH)_2_ and represents the first basic strontium oxotellurate(IV), *viz*. with the presence of an OH functionality. In comparison with the other strontium oxotellurates(IV) compiled in Section 3, all Sr—O and Te—O lengths are in similar ranges.

## Database survey   

In the Inorganic Crystal Structure Database (ICSD, 2016 ▸) structural data for the following hydrous or anhydrous strontium oxotellurate(IV) phases have been deposited: SrTe_5_O_11_ (Burckhardt & Trömel, 1983 ▸), Sr_3_Te_4_O_11_ (Dytyatyev & Dolgikh, 1999 ▸), various polymorphs of SrTeO_3_ (Dityatiev *et al.*, 2006 ▸; Zavodnik *et al.*, 2007*a* ▸,*b* ▸,*c* ▸, 2008 ▸; Stöger *et al.*, 2011 ▸), SrTe_3_O_8_ (Barrier *et al.*, 2006 ▸; Weil & Stöger, 2007 ▸) and SrTeO_3_(H_2_O) (Stöger *et al.*, 2011 ▸). Additionally, in the Inter­national Centre for Diffraction Data PDF-4 database (ICDD, 2015 ▸) diffraction data for the following phases are compiled: Sr_2_Te_3_O_8_ (Elerman & Koçak, 1986 ▸), SrTe_2_O_5_ (Redman *et al.*, 1970 ▸; Gorbenko *et al.*, 1983 ▸) and a high-temperature phase of the latter (Külcü *et al.*, 1984 ▸).

## Synthesis and crystallization   

For the hydro­thermal experiment, a Teflon container was filled with 0.0733 g of strontium oxide, 0.1529 g of tellurium dioxide and 0.032 ml of selenic acid (conc.; 96 wt%), corresponding to the stoichiometric ratio 3:2:1. To this mixture 10 ml water were added to about three-fourth of the container volume. The container was then sealed with a Teflon lid and loaded into a stainless steel autoclave and then heated at autogenous pressure in an oven at 403 K for one week. After the reaction time, the autoclave was allowed to cool down to room temperature over six h. The formed solid product was filtered off and washed with water and ethanol. Inspection under a polarizing microscope revealed a phase mixture with different crystal forms clearly discernible. According to X-ray powder diffraction of the bulk material, the following phases could be identified: α-TeO_2_ (Lindqvist, 1968 ▸), SrTe_2_O_5_ (Redman *et al.*, 1970 ▸), SrTe_3_O_8_ (Barrier *et al.*, 2006 ▸; Weil & Stöger, 2007 ▸) and SrTe_5_O_11_ (Burckhardt & Trömel, 1983 ▸). Solid reaction products containing Se-phases were not detected. Platy Sr_5_Te_4_O_12_(OH)_2_ crystals were present in only minor amounts, and were manually separated for structure determination from the other solid products.

## Refinement   

Crystal data, data collection and structure refinement details are summarized in Table 4 ▸. Some of the O atoms showed physically unreasonable behaviour when refined with anisotropic displacement parameters. Hence, for the final model all O atoms were refined with individual isotropic displacement parameters. The H atom of the OH group (or positionally disordered parts) could not be located and thus was not included in the model. Twinning by inversion was also taken into account, with a contribution of the minor twin component of about 6%. The maximum and minimum remaining electron densities are found 2.34 and 0.96 Å, respectively, from Sr3.

## Supplementary Material

Crystal structure: contains datablock(s) I, global. DOI: 10.1107/S2056989016015577/hb7619sup1.cif

Structure factors: contains datablock(s) I. DOI: 10.1107/S2056989016015577/hb7619Isup2.hkl

CCDC reference: 1508062

Additional supporting information: crystallographic information; 3D view; checkCIF report

## Figures and Tables

**Table 1:** Selected geometric parameters (Å, °)

Sr1—O7	2.430 (12)	Sr3—O2	2.507 (11)
Sr1—O5^i^	2.476 (12)	Sr3—O4^v^	2.517 (11)
Sr1—O1^ii^	2.593 (12)	Sr3—O6^i^	2.536 (11)
Sr1—O3	2.596 (11)	Sr3—O6^vi^	2.590 (12)
Sr1—O2^iii^	2.616 (12)	Sr3—O4^vii^	2.644 (11)
Sr1—O7^iii^	2.700 (11)	Sr3—O1^viii^	2.666 (11)
Sr1—O2	2.852 (12)	Te1—O6	1.865 (11)
Sr2—O3	2.510 (11)	Te1—O2	1.871 (11)
Sr2—O1^iv^	2.624 (12)	Te1—O5	1.890 (12)
Sr2—O5	2.633 (12)	Te2—O4	1.858 (11)
Sr2—O7	2.960 (11)	Te2—O3	1.882 (11)
Sr3—O7^iii^	2.393 (11)	Te2—O1	1.886 (11)
			
O6—Te1—O2	99.4 (4)	O4—Te2—O3	101.1 (5)
O6—Te1—O5	100.3 (5)	O4—Te2—O1	100.3 (5)
O2—Te1—O5	98.0 (5)	O3—Te2—O1	98.8 (5)

Symmetry codes: (i) 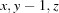; (ii) 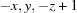; (iii) 
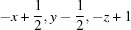
; (iv) 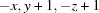; (v) 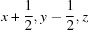; (vi) 
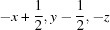
; (vii) 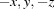; (viii) 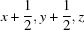.

**Table 2:** Results of the bond-valance-sum (BVS) analysis

Atom	BVS	Δ to expected value
Sr1	2.07	0.07
Sr2	1.91	0.09
Sr3	2.23	0.23
Te1	3.94	0.06
Te2	3.93	0.07
O1	2.04	0.04
O2	2.08	0.08
O3	1.91	0.09
O4	1.96	0.04
O5	1.89	0.11
O6	1.95	0.05
O7	1.21	0.79

BVS parameters of Brown & Altermatt (1985 ▸) were used for all bonds.

**Table 3:** Hydrogen-bond geometry (Å)

*D*—H⋯*A*	*D*⋯*A*
O7⋯O5	2.808 (12)
O7⋯O2	2.893 (12)
O7⋯O2^ix^	2.991 (11)
O7⋯O1^iv^	3.063 (11)

Symmetry codes: (iv) 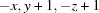; (ix) 
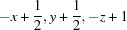
.

**Table 4:** Experimental details

Crystal data	
Chemical formula	Sr_5_Te_4_O_12_(OH)_2_
*M*_r_	1174.52
Crystal system, space group	Monoclinic, *C*2
Temperature (K)	295
*a*, *b*, *c* (Å)	16.0785 (10), 5.7927 (5), 8.9262 (7)
β (°)	107.542 (4)
*V* (Å^3^)	792.71 (11)
*Z*	2
Radiation type	Mo *K*α
μ (mm^−1^)	23.99
Crystal size (mm)	0.18 × 0.06 × 0.01

Data collection	
Diffractometer	Bruker APEXII CCD
Absorption correction	Multi-scan (*SADABS*; Bruker, 2012 ▸)
*T*_min_, *T*_max_	0.099, 0.795
No. of measured, independent and observed [*I* > 2σ(*I*)] reflections	12913, 1914, 1319
*R*_int_	0.088
(sin θ/λ)_max_ (Å^−1^)	0.660

Refinement	
*R*[*F* ^2^ > 2σ(*F* ^2^)], *wR*(*F* ^2^), *S*	0.042, 0.085, 1.02
No. of reflections	1914
No. of parameters	71
No. of restraints	1
H-atom treatment	H-atom parameters not defined
Δρ_max_, Δρ_min_ (e Å^−3^)	2.31, −1.78
Absolute structure	Refined as an inversion twin
Absolute structure parameter	0.058 (18)

Computer programs: *APEX2* and *SAINT* (Bruker, 2012 ▸), *SHELXS97* (Sheldrick, 2008 ▸), *SHELXL2014* (Sheldrick, 2015 ▸), *ATOMS* (Dowty, 2006 ▸) and *publCIF* (Westrip, 2010 ▸).

## supplementary crystallographic information

### Crystal data

**Table:**

Sr_5_Te_4_O_12_(OH)_2_	*F*(000) = 1024
*M**_r_* = 1174.52	*D*_x_ = 4.921 Mg m^−^^3^
Monoclinic, *C*2	Mo *K*α radiation, λ = 0.71073 Å
Hall symbol: C 2y	Cell parameters from 2028 reflections
*a* = 16.0785 (10) Å	θ = 4.2–33.0°
*b* = 5.7927 (5) Å	µ = 23.99 mm^−^^1^
*c* = 8.9262 (7) Å	*T* = 295 K
β = 107.542 (4)°	Plate, colourless
*V* = 792.71 (11) Å^3^	0.18 × 0.06 × 0.01 mm
*Z* = 2	

### Data collection

**Table:**

Bruker APEXII CCD diffractometer	1319 reflections with *I* > 2σ(*I*)
Radiation source: fine-focus sealed tube	*R*_int_ = 0.088
ω and φ scans	θ_max_ = 28.0°, θ_min_ = 2.4°
Absorption correction: multi-scan (*SADABS*; Bruker, 2012)	*h* = −20→21
*T*_min_ = 0.099, *T*_max_ = 0.795	*k* = −7→7
12913 measured reflections	*l* = −11→11
1914 independent reflections	

### Refinement

**Table:**

Refinement on *F*^2^	H-atom parameters not defined
Least-squares matrix: full	*w* = 1/[σ^2^(*F*_o_^2^) + (0.0236*P*)^2^ + 0.7139*P*] where *P* = (*F*_o_^2^ + 2*F*_c_^2^)/3
*R*[*F*^2^ > 2σ(*F*^2^)] = 0.042	(Δ/σ)_max_ < 0.001
*wR*(*F*^2^) = 0.085	Δρ_max_ = 2.31 e Å^−^^3^
*S* = 1.02	Δρ_min_ = −1.78 e Å^−^^3^
1914 reflections	Absolute structure: Refined as an inversion twin
71 parameters	Absolute structure parameter: 0.058 (18)
1 restraint	

### Special details

**Table:**

Geometry. All esds (except the esd in the dihedral angle between two l.s. planes) are estimated using the full covariance matrix. The cell esds are taken into account individually in the estimation of esds in distances, angles and torsion angles; correlations between esds in cell parameters are only used when they are defined by crystal symmetry. An approximate (isotropic) treatment of cell esds is used for estimating esds involving l.s. planes.
Refinement. Refined as a 2-component inversion twin

### Fractional atomic coordinates and isotropic or equivalent isotropic displacement parameters (Å^2^)

**Table:**

	*x*	*y*	*z*	*U*_iso_*/*U*_eq_	
Sr1	0.16911 (10)	0.0360 (4)	0.4988 (2)	0.0097 (5)	
Sr2	0.0000	0.5471 (4)	0.5000	0.0094 (8)	
Sr3	0.27399 (11)	0.0613 (2)	0.1447 (2)	0.0071 (5)	
Te1	0.10820 (7)	0.52512 (19)	0.16455 (15)	0.0081 (3)	
Te2	−0.05011 (7)	0.0246 (2)	0.17800 (14)	0.0091 (3)	
O1	−0.0998 (7)	−0.1557 (19)	0.3050 (14)	0.015 (3)*	
O2	0.2040 (8)	0.3491 (18)	0.2785 (14)	0.017 (3)*	
O3	0.0218 (7)	0.2152 (19)	0.3350 (14)	0.013 (3)*	
O4	−0.1423 (7)	0.2229 (19)	0.0869 (14)	0.018 (3)*	
O5	0.0964 (7)	0.7088 (19)	0.3320 (14)	0.017 (3)*	
O6	0.1651 (7)	0.7327 (19)	0.0690 (14)	0.014 (3)*	
O7	0.1881 (7)	0.4343 (18)	0.5881 (13)	0.017 (3)*	

### Atomic displacement parameters (Å^2^)

**Table:**

	*U*^11^	*U*^22^	*U*^33^	*U*^12^	*U*^13^	*U*^23^
Sr1	0.0090 (8)	0.0107 (11)	0.0086 (10)	−0.0015 (8)	0.0013 (7)	−0.0017 (9)
Sr2	0.0108 (13)	0.006 (2)	0.0134 (14)	0.000	0.0063 (10)	0.000
Sr3	0.0055 (8)	0.0035 (13)	0.0127 (10)	−0.0001 (7)	0.0036 (6)	−0.0012 (9)
Te1	0.0066 (6)	0.0084 (8)	0.0087 (6)	0.0003 (6)	0.0014 (4)	0.0000 (6)
Te2	0.0092 (6)	0.0062 (7)	0.0126 (7)	−0.0013 (7)	0.0041 (4)	−0.0024 (8)

### Geometric parameters (Å, º)

**Table:**

Sr1—O7	2.430 (12)	Sr3—O2	2.507 (11)
Sr1—O5^i^	2.476 (12)	Sr3—O4^vi^	2.517 (11)
Sr1—O1^ii^	2.593 (12)	Sr3—O6^i^	2.536 (11)
Sr1—O3	2.596 (11)	Sr3—O6^vii^	2.590 (12)
Sr1—O2^iii^	2.616 (12)	Sr3—O4^viii^	2.644 (11)
Sr1—O7^iii^	2.700 (11)	Sr3—O1^ix^	2.666 (11)
Sr1—O2	2.852 (12)	Te1—O6	1.865 (11)
Sr2—O3	2.510 (11)	Te1—O2	1.871 (11)
Sr2—O3^ii^	2.510 (11)	Te1—O5	1.890 (12)
Sr2—O1^iv^	2.624 (12)	Te2—O4	1.858 (11)
Sr2—O1^v^	2.624 (11)	Te2—O3	1.882 (11)
Sr2—O5	2.633 (12)	Te2—O1	1.886 (11)
Sr2—O5^ii^	2.633 (12)	O7—O1^iv^	3.063 (11)
Sr2—O7^ii^	2.960 (11)	O7—O2	2.893 (12)
Sr2—O7	2.960 (11)	O7—O2^x^	2.991 (11)
Sr3—O7^iii^	2.393 (11)	O7—O5	2.808 (12)
			
O7—Sr1—O5^i^	156.1 (4)	O2—Sr3—O6^i^	104.7 (4)
O7—Sr1—O1^ii^	102.8 (4)	O4^vi^—Sr3—O6^i^	74.3 (3)
O5^i^—Sr1—O1^ii^	81.8 (4)	O7^iii^—Sr3—O6^vii^	144.4 (4)
O7—Sr1—O3	79.0 (4)	O2—Sr3—O6^vii^	114.8 (4)
O5^i^—Sr1—O3	77.4 (4)	O4^vi^—Sr3—O6^vii^	78.7 (4)
O1^ii^—Sr1—O3	92.7 (4)	O6^i^—Sr3—O6^vii^	118.4 (3)
O7—Sr1—O2^iii^	98.7 (4)	O7^iii^—Sr3—O4^viii^	142.3 (4)
O5^i^—Sr1—O2^iii^	105.0 (4)	O2—Sr3—O4^viii^	76.6 (4)
O1^ii^—Sr1—O2^iii^	72.8 (4)	O4^vi^—Sr3—O4^viii^	117.8 (3)
O3—Sr1—O2^iii^	164.5 (4)	O6^i^—Sr3—O4^viii^	74.4 (3)
O7—Sr1—O7^iii^	105.5 (3)	O6^vii^—Sr3—O4^viii^	71.3 (3)
O5^i^—Sr1—O7^iii^	87.0 (4)	O7^iii^—Sr3—O1^ix^	74.3 (4)
O1^ii^—Sr1—O7^iii^	132.6 (4)	O2—Sr3—O1^ix^	73.4 (4)
O3—Sr1—O7^iii^	129.5 (4)	O4^vi^—Sr3—O1^ix^	102.8 (3)
O2^iii^—Sr1—O7^iii^	65.9 (4)	O6^i^—Sr3—O1^ix^	163.2 (4)
O7—Sr1—O2	65.9 (4)	O6^vii^—Sr3—O1^ix^	76.3 (3)
O5^i^—Sr1—O2	103.2 (4)	O4^viii^—Sr3—O1^ix^	120.2 (3)
O1^ii^—Sr1—O2	162.2 (4)	O6—Te1—O2	99.4 (4)
O3—Sr1—O2	72.1 (3)	O6—Te1—O5	100.3 (5)
O2^iii^—Sr1—O2	121.1 (2)	O2—Te1—O5	98.0 (5)
O7^iii^—Sr1—O2	65.2 (3)	O4—Te2—O3	101.1 (5)
O3—Sr2—O3^ii^	80.0 (5)	O4—Te2—O1	100.3 (5)
O3—Sr2—O1^iv^	136.6 (4)	O3—Te2—O1	98.8 (5)
O3^ii^—Sr2—O1^iv^	106.2 (3)	Te2—O1—Sr1^ii^	121.0 (5)
O3—Sr2—O1^v^	106.2 (3)	Te2—O1—Sr2^i^	118.5 (5)
O3^ii^—Sr2—O1^v^	136.6 (4)	Sr1^ii^—O1—Sr2^i^	97.7 (4)
O1^iv^—Sr2—O1^v^	98.0 (5)	Te2—O1—Sr3^xi^	114.2 (5)
O3—Sr2—O5	74.2 (3)	Sr1^ii^—O1—Sr3^xi^	102.3 (4)
O3^ii^—Sr2—O5	144.8 (4)	Sr2^i^—O1—Sr3^xi^	99.6 (4)
O1^iv^—Sr2—O5	78.3 (4)	Te1—O2—Sr3	121.2 (6)
O1^v^—Sr2—O5	74.7 (3)	Te1—O2—Sr1^x^	120.9 (5)
O3—Sr2—O5^ii^	144.8 (4)	Sr3—O2—Sr1^x^	106.2 (4)
O3^ii^—Sr2—O5^ii^	74.2 (3)	Te1—O2—Sr1	114.9 (5)
O1^iv^—Sr2—O5^ii^	74.7 (3)	Sr3—O2—Sr1	96.6 (3)
O1^v^—Sr2—O5^ii^	78.3 (4)	Sr1^x^—O2—Sr1	90.5 (4)
O5—Sr2—O5^ii^	138.3 (5)	Te2—O3—Sr2	136.3 (5)
O3—Sr2—O7^ii^	89.3 (3)	Te2—O3—Sr1	115.9 (5)
O3^ii^—Sr2—O7^ii^	71.0 (3)	Sr2—O3—Sr1	103.9 (4)
O1^iv^—Sr2—O7^ii^	133.8 (3)	Te2—O4—Sr3^xii^	142.8 (6)
O1^v^—Sr2—O7^ii^	66.2 (3)	Te2—O4—Sr3^viii^	117.9 (5)
O5—Sr2—O7^ii^	131.1 (3)	Sr3^xii^—O4—Sr3^viii^	94.8 (4)
O5^ii^—Sr2—O7^ii^	60.0 (3)	Te1—O5—Sr1^v^	139.9 (6)
O3—Sr2—O7	71.0 (3)	Te1—O5—Sr2	117.8 (5)
O3^ii^—Sr2—O7	89.3 (3)	Sr1^v^—O5—Sr2	100.5 (4)
O1^iv^—Sr2—O7	66.2 (3)	Te1—O6—Sr3^v^	139.1 (6)
O1^v^—Sr2—O7	133.8 (3)	Te1—O6—Sr3^xiii^	115.9 (5)
O5—Sr2—O7	60.0 (3)	Sr3^v^—O6—Sr3^xiii^	95.7 (4)
O5^ii^—Sr2—O7	131.1 (3)	Sr3^x^—O7—Sr1	126.0 (5)
O7^ii^—Sr2—O7	154.5 (4)	Sr3^x^—O7—Sr1^x^	103.7 (4)
O7^iii^—Sr3—O2	75.2 (4)	Sr1—O7—Sr1^x^	98.5 (4)
O7^iii^—Sr3—O4^vi^	88.5 (4)	Sr3^x^—O7—Sr2	97.4 (4)
O2—Sr3—O4^vi^	163.7 (4)	Sr1—O7—Sr2	96.0 (4)
O7^iii^—Sr3—O6^i^	89.0 (4)	Sr1^x^—O7—Sr2	139.9 (4)

Symmetry codes: (i) *x*, *y*−1, *z*; (ii) −*x*, *y*, −*z*+1; (iii) −*x*+1/2, *y*−1/2, −*z*+1; (iv) −*x*, *y*+1, −*z*+1; (v) *x*, *y*+1, *z*; (vi) *x*+1/2, *y*−1/2, *z*; (vii) −*x*+1/2, *y*−1/2, −*z*; (viii) −*x*, *y*, −*z*; (ix) *x*+1/2, *y*+1/2, *z*; (x) −*x*+1/2, *y*+1/2, −*z*+1; (xi) *x*−1/2, *y*−1/2, *z*; (xii) *x*−1/2, *y*+1/2, *z*; (xiii) −*x*+1/2, *y*+1/2, −*z*.

### Hydrogen-bond geometry (Å)

**Table:**

*D*—H···*A*	*D*···*A*
O7···O5	2.808 (12)
O7···O2	2.893 (12)
O7···O2^x^	2.991 (11)
O7···O1^iv^	3.063 (11)

Symmetry codes: (iv) −*x*, *y*+1, −*z*+1; (x) −*x*+1/2, *y*+1/2, −*z*+1.
